# Voices as Cues to Children’s Needs for Caregiving

**DOI:** 10.1007/s12110-021-09418-4

**Published:** 2021-12-09

**Authors:** Carlos Hernández Blasi, David F. Bjorklund, Sonia Agut, Francisco Lozano Nomdedeu, Miguel Ángel Martínez

**Affiliations:** 1 Departamento de Psicología, Universitat Jaume I, 12071 Castellón, Spain; 2 Florida Atlantic University, Boca Raton, FL USA

**Keywords:** Voice, Caregiving, Early childhood, Evolutionary developmental psychology

## Abstract

The aim of this study was to explore the role of voices as cues to adults of children’s needs for potential caregiving during early childhood. To this purpose, 74 college students listened to pairs of 5-year-old versus 10-year-old children verbalizing neutral-content sentences and indicated which voice was better associated with each of 14 traits, potentially meaningful in interactions between young children and adults. Results indicated that children with immature voices were perceived more positively and as being more helpless than children with mature voices. Children’s voices, regardless of the content of speech, seem to be a powerful source of information about children’s need for caregiving for parents and others during the first six years of life.

Caregiving in humans is a universal characteristic with a long evolutionary history (Lang & Fowers, 2019). Indeed, it has been suggested that caregiving is a primate adaptation (de Waal, 1996; Warneken & Tomasello, 2006). Caregiving is critical for children’s survival, particularly in infancy, but also during early childhood (between the ages of about 3 and 7 years) and beyond. Accordingly, human adults seem to have developed a series of adaptations in order to provide proper care to vulnerable offspring: (a) a sensitivity to neotenous cues and distress vocalizations; (b) specific tactile behaviors such as skin-to-skin contact; (c) attachment-related behaviors; and (d) the possibility of experiencing compassionate affect when faced with infants’ suffering (Goetz et al., 2010). For their part, children seem to have evolved simultaneously a series of adaptations to capture adults’ attention and convey their needs and emotions, what Trivers (1974:249) referred to as “psychological weapons in order to compete with their parents.” Thus, parenting involves a series of complex cost–benefit decisions wherein both adults’ and children’s inclusive fitness and behaviors guide final parental investment (Lancaster et al., 2010). Two well-studied examples of such “psychological weapons” in infancy are the organization of facial features and crying.

Ethologist Konrad Lorenz (1943) was the first to suggest that typical infant facial features, including large, rounded cheeks, a flat nose, rounded head, large head relative to body size, and adult-sized eyes (the “infant schema,” or *Kindchenschema*), might serve as an innate releasing mechanism that promotes positive adult caretaking behaviors. Subsequent research has generally confirmed this view (e.g., Franklin & Volk, 2018; Glocker et al., 2009; Senese et al., 2013), with allure toward cute infant faces being present in adolescence, and even in childhood (Borgi et al., 2014; Fullard & Reiling, 1976; Luo et al., 2020), mediated by both hormonal and neural systems (Kringelbach et al., 2016; Luo et al., 2015).

Crying is another extensively studied infant adaptation. It serves to maintain proximity with potential caregivers and to guide caregivers’ behavior (Bowlby, 1969; Soltis, 2004). Indeed, acoustic properties of crying convey critical information about infants’ needs and emotions (e.g., Wolff, 1969) and physical condition (e.g., Furlow, 1997). Crying particularly draws parents’ attention when it is different from the typical infants’ crying (Chittora & Patil, 2017), and it has been suggested to be a focal point for regulating parental investment (e.g., De Vries, 1984) and maltreatment (e.g., Frodi, 1985). Fathers seem to be as good as mothers at recognizing their babies’ cries, if they have spent enough time with them (Gustafsson et al., 2013), and, curiously enough, it has been found that infants adjust their crying melody to their native language. For example, fundamental frequency (F0) or pitch contour (melody) of crying is different in French and German newborns, shaping the melody of their respective mother languages (Mampe et al., 2009). Similar differences have also been reported between German, Chinese, and Nso (Cameroon) infants’ crying (Wermke et al., 2016, 2017).

## Cues Signaling Need for Care during Early Childhood

In contrast to infancy, we know much less about the potential cues signalling the need for care expressed by children during early childhood. This is somewhat surprising given that children are still highly vulnerable following weaning (typically at the age of about 3 years, on average, in traditional societies; Dettwyler, 2017), and, in most traditional societies, they require a long period of intensive allomaternal care (e.g., Konner, 2010; Lancy, 2015). We do know that facial cues do not seem to be as important for promoting caregiving during early childhood as they are during infancy. For example, according to adults’ judgments, 4.5-year-old children’s faces do not differ significantly in attractiveness and likeability from those of adults (Luo et al., 2011). However, the remarkable improvement in language skills following infancy increases the relevance of children’s speech as cues for the need for care. In this vein, children who verbalize certain types of immature explanations of ordinary phenomena (what has been called “supernatural thinking”: e.g., “The sun’s not out today because it’s mad,” “The big peak is for long walks, and the small peak is for short walks”) are perceived more positively and helpless by both adults and older adolescents (14 to 17 years old) than children verbalizing more mature, adult-like explanations of the same phenomena (e.g., “The sun’s not out today because the clouds are blocking it”) (Bjorklund et al., 2010; Periss et al., 2012). In addition, these cues of immature thinking have been shown to prevail over physical cues (e.g., faces) in both adults and older adolescents when both are available (Hernández Blasi & Bjorklund, 2018; Hernández Blasi et al., 2015, 2017). However, little is known yet, to our knowledge, about the potential role of the voice (vocal cues) as a cue for the need for careging during early childhood, and this is the main purpose of the present study.

## Vocalizations as Cues of Immaturity

Vocal communication is ubiquitous in mammals, birds, amphibians, and reptiles (Hauser, 1996; Titze, 2017), dating back perhaps 30 million years (Belin et al., 2011). Quite probably, early nonhuman primates and our most direct hominin ancestors used it to convey long-distance information about dangers (alarm calls) and opportunities, signalling and maintaining dominance, and finding mates (Cook, 2002; Seyfarth & Cheney, 2003). In humans, vocal communication seems to have preceded the evolution of speech (Cook, 2002; Titze, 2017). Voices are indeed special for the human brain (Belin 2011), with some voice-selective neuron populations already present in 7-month-old infants (Grossman et al., 2010).

Human voices vary on a series of parameters, such as pitch, intensity, and timbre. Pitch, as expressed by fundamental frequency (F0) (an acoustic property linked to the vibration rate of vocal folds during phonation, measured in Hertz, Hz), is likely one of the most salient and empirically studied parameters (Cook, 2002; Rosenfield et al., 2020; Titze, 2000). Significant changes in pitch take place during early childhood, puberty, and later adulthood, often driven by hormonal changes (Titze, 2000). Newborns’ crying has a fundamental frequency between 400 and 600 Hz, whereas 3- to 6-years-olds’ fundamental frequency averages about 265 Hz (e.g., Capellari & Cielo, 2008; Michelsson & Michelsson, 1999; Trollinger, 2003). Sex differences in children’s voices can be identified beginning by about 4 years of age, although not always accurately, particularly for girls (e.g., Karlsson & Rothenberg, 1987; Perry et al., 2001; Sergeant et al., 2005). From 7 to 17 years of age, fundamental frequency lowers on average from about 250 Hz to about 200 Hz in females and to about 125 Hz in males, with the greatest decrease occurring in boys at 13–14 years of age (e.g., Balasubramaniam & Nikhita, 2017; Berger et al., 2019; Schneider et al., 2010).

Adults estimate children’s age relatively well based on their voices until children are about 11 years of age, and they then systematically tend to underestimate the age of older girls (Assmann et al., 2013). Adults identify children’s sex better as children get older, although they are more accurate for older boys than for older girls (Assmann et al., 2011), and height is more precisely predicted from voice when the sex of the child is known—for instance, when an older girl is misidentified as a boy, her height is typically underestimated (Assmann et al., 2018). Adults’ ability to estimate the age of young children relatively accurately on the basis of their natural voices may have made differences in children’s voices a good target for natural selection to use as a cue to immaturity and the need for care.

## The Current Study

The current study assessed the potential role of children’s voices as cues for needing care during early childhood (between the ages of about 3 and 7 years) by exploring how adults’ perception of some children’s traits can be inferred from the maturity of their voices. To this purpose, we presented samples of voices of both preschool (about 5 years old) and school-age (about 10 years old) children to groups of college students and asked their impressions about the degree of positive affect, negative affect, intelligence, and helplessness evoked by the voices. We also measured participants’ reaction times to make their decisions.

The samples of voices were recorded in natural settings while children verbalized neutral-content sentences. We hypothesized, first, that children with immature voices would be perceived by adults as having more positive affect and being more helpless than children with mature voices. Based on earlier research on adults’ perception of children’s verbalized thinking and facial features using the same paradigm (e.g., Bjorklund et al., 2010; Hernández Blasi et al., 2015), we also predicted that there would likely be no significant differences on negative-affect ratings between children with immature and those with mature voices. Finally, we made no predictions about adults’ reactions regarding items reflecting intelligence. On the one hand, one might expect, according to previous literature indicating that adults can accurately estimate children’s age based on their voices (e.g., Assmann et al., 2013), that children with mature voices would be identified as older and thus be more apt to be selected on intelligence items than children with immature voices. On the other hand, research has also shown that children with mature faces are not always considered as more intelligent than children with immature faces (e.g., Hernández Blasi & Bjorklund, 2018), and this could possibly be the case for voices.

Second, we anticipated longer reaction times for the Negative-Affect items than for the other trait dimensions given that, as shown in previous research (e.g., Hernández Blasi et al., 2017), adults have more difficulty (i.e., take longer to make a decision) when having to assess children on negative as opposed to positive items. We did not make any predictions about which trait dimension (Positive Affect, Intelligence, Helpless), if any, would likely be the easiest for participants in terms of speed of decision-making.

## Method

### Participants

The sample consisted of 74 adults (61 female, *M*_age_ = 21.6 years, *SD* = 6 years, age range = 17–54 years) attending a public urban university in eastern Spain. All participants were college students, most taking classes in psychology (60, 81%), with the remainder taking classes in other degrees (e.g., education) or educational levels (e.g., master’s). Their socioeconomic background was mainly middle class, typical in public universities in Spain. Participants were tested individually at the researchers’ laboratory. All participants volunteered for this study and received a small monetary compensation (2 euros). The study was approved by the University Research Ethics Committee.

### Design

To obtain samples of the children’s voices, 53 children aged 3 to 12 years old (26 boys and 27 girls) were recorded at their school with parental permission. Children were audiorecorded individually in a small and relatively isolated and noise-free classroom using a TASCAM DR-40 digital recorder. After establishing rapport, we asked children to repeat two practice sentences: (1) “Today we are at the school ‘Grans i Menuts’” and (2) “My name is [child’s given name] and your name is [researcher’s given name].” Each child was then asked to repeat four neutral-content sentences, sequentially read aloud by one of the experimenters: (1) “I like the beach more than the mountains,” (2) “I like the mountains more than the beach,” (3) “I like traveling more by plane than by car,” and (4) “I like travelling more by car than by plane.” (These are English translations of the Spanish sentences that were recorded.) When a problem was detected (e.g., a pronunciation problem; voice was too low; a change in the words of the sentence), we asked children to repeat the sentence.

Edits of the four sentences for each child were made using the free open-source audio-editor Audacity (version 2.1.0). Measures of the pitch (fundamental frequency, Hz) and intensity (volume, dB) of the children’s voices were taken by means of Praat (version 6.0.24), a free-access software for phonetic speech analysis, designed by Paul Boersma and David Weenink from the University of Amsterdam. Recordings of four boys and four girls were selected (four 5- and four 10-year-olds), and four different sets of sentences were generated. Each set contained the eight children and the four neutral sentences, with four pairs of a 5- versus a 10-year-old child of the same sex verbalizing the same sentence. However, in each set, each of the four neutral sentences was articulated by a different pair of children, such that each pair of children verbalized the four neutral sentences across the four sets, but a different one per set.^1^

Table 1 presents mean pitch (fundamental frequency, Hz) for 5- and 10-year-old boys’ and girls’ voices across the four sets. Intensity (volume, dB) of all the voice samples was equalized to about 72 dB volume, which, according to some voice experts (e.g., Bustos, 2012), would be within the typical range for spoken voice in Spain (65 to 75 dB). More specific criteria used for selecting the vocal stimuli are described in Appendix 2.

**Table 1:** Mean pitch (fundamental frequency, in Hz) of the boys’ and girls’ voices, across the four neutral sentences verbalized in every set. (standard deviations in parenthesis)

	Boys	Girls
5-year-old	294.15 (26.07)	286.68 (10.94)
10-year-old	213.16 (15.13)	252.79 (10.30)

We used E-prime (version 2.0) professional software to implement our design into a computerized experimental protocol in a way that allowed us to obtain data on participants’ decisions and reaction times. Within every set, the order of presentation of each pair of voices was counterbalanced. The order of presentation of the 10-year-old voices and the 5-year-old voices in each pair was also counterbalanced across sets.

### Procedure

Participants were tested individually at a university laboratory. An experimenter explained the procedure to the participant, who was assigned to one of the four sets generated for each condition. Birthdate, sex, and university degree program were the only personal data collected from the participants. The experiment was presented on an Acer V193 HQV LCD 18.5″ wide monitor, and the auditory stimuli were played through SHURE SRH440 adjustable headphones. Following completion of the study, which lasted between 5 and 10 min (although there was no time limit to complete the experiment), participants were thanked and given 2 euros.

The participants first read instructions about the experimental procedure on the computer screen (“Now you will listen to some short sentences. After listening to them, press any key [of the keyboard] and a series of questions will show up that you will have to answer one by one.”). Participants were told they should (1) press the key with a yellow sticker on it (over the letter Z of a QWERTY keyboard) to select the child whose voice was associated with an icon on the left side of the screen or (2) press the key with a green sticker on it (over the letter M) to select the child whose voice was associated with an icon on the right side of the screen. Participants were then told that they could press one of the two keys with red stickers on them (over the numbers 1 and 0) to listen again to either of the children’s voices. The participant was then presented with two practice trials (one with a pair of boys’ voices, and another with a pair of girls’ voices) to become familiar with the procedure.

**Fig. 1:**
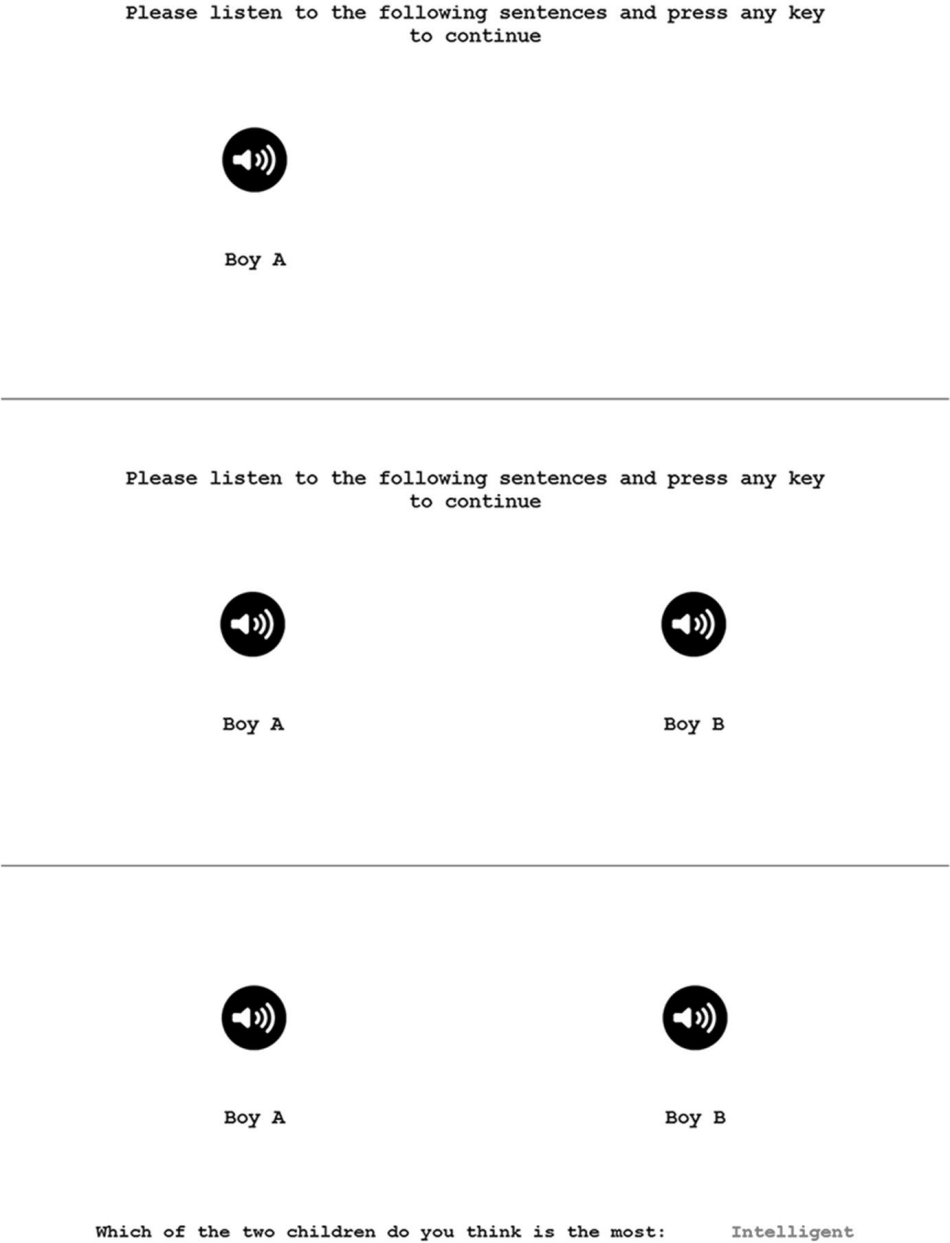
Core audiovisual sequence presented to participants for this experiment on the computer screen. *Screen 1:* Five seconds after the instruction at the top of the screen appeared, an icon emerged from the middle-left side of the screen with a “Boy A” or “Girl A” indicated below, and then a neutral sentence (e.g., uttered by a 5-year-old child) was presented. *Screen 2:* One second later the second icon with a “Boy B” or “Girl B” indicated below appeared, and the same neutral sentence uttered by the other child of the pair (in this example, a 10-year-old child) was presented. *Screen 3:* After the participant pressed a key on the keyboard, the first question appeared at the bottom of the screen; once the first question was answered, 13 more questions with their corresponding 13 adjectives or short statements were delivered sequentially, in random order, at the same screen place

Following practice, the participant was presented with four new pairs of children’s voices (two pairs of boys and two pairs of girls) appearing sequentially. Every pair presentation started with a short instruction printed at the upper side of the screen (Fig. 1): “Please listen to the following sentences and press any key to continue.” Five seconds later an icon consisting of a small black circle with a white speaker inside appeared from the middle-left side of the screen with a written indication below (e.g., “Boy A” or “Girl A”), and the first neutral sentence (e.g., uttered by a 5-year-old child) was presented. One second later, the same audiovisual sequence was repeated for the second child of the pair (e.g., a 10-year-old child), this time with the icon appearing from the middle-right side of the screen (e.g., “Boy B” or “Girl B”), in parallel and at the same height as the previous one. The two children (A and B) verbalized exactly the same neutral sentence. Once the participant had listened to the two sentences and pressed a key on the keyboard, a written question appeared from the bottom of the screen: “Which of the two children do you think is the most [adjective or short statement in red]?” After the first question was answered, 13 more questions with their corresponding 13 adjectives or short statements were delivered sequentially. Then a new pair of children, verbalizing the next neutral sentence, was presented on the screen following the same procedure. The order of presentation of the 14 adjectives or short statements was systematically and automatically counterbalanced by the computer program.

These 14 adjectives and short statements have been used in previous research (e.g., Bjorklund et al., 2010; Hernández Blasi et al., 2017) and constitute a selection of traits that are potentially meaningful in understanding interactions between adults and young children. Based on principal component analyses performed in these previous studies, we grouped the items into four factors or trait dimensions: Positive Affect (*cute*, *friendly*, *nice*, *likeable*), Negative Affect (*sneaky*, *likely to lie*, *feel more irritated with*, *feel more angry with*), Intelligence (*smart*, *intelligent*), and Helpless (*helpless*, *feel more protective towards*, *feel like helping*). One item (*curious*) did not load highly on any factor, and it was not included in subsequent analyses.

## Results

When participants selected 5-year-old children for an adjective or short statement, their response was coded as 1, and when they selected 10-year-old children, their response was coded as 0. Therefore, mean scores significantly greater than 0.5 reflect that participants selected the 5-year-old children’s voices more often, whereas mean scores significantly less than 0.5 indicate that participants selected the 10-year-old children’s voices more often. Table 2 presents the proportion of participants who selected the 5-year-old (immature) voices by trait dimension (Positive Affect, Negative Affect, Intelligence, Helpless), as well as the mean reaction time per item.

**Table 2:** Proportion of participants selecting the 5-year-old child, and mean reaction time (in milliseconds) by trait dimension (Positive Affect, Negative Affect, Intelligence, Helpless) (standard deviations in parenthesis)

	Positive Affect (*n* = 4)	Negative Affect (*n* = 4)	Intelligence (*n* = 2)	Helpless (*n* = 3)
Proportion	.62^*a*^ (.18)	.51 (.27)	.19^*b*^ (.23)	.85^*a*^ (.19)
Reaction Time	2162.49 (711.42)	2478.35 (874.42)	2069.71 (771.50)	2002.00 (613.13)

^*a*^ selecting the 5-year-old child significantly greater than expected by chance; ^*b*^ selecting the 10-year-old child significantly greater than expected by chance. Significance set at *p* < .001

To analyze mean scores we first applied a series of two-tailed, single-sample *t*-tests (*p* < 0.001 to adjust for multiple contrasts) to determine whether the 5-year-old or the 10-year-old children’s voices were selected significantly greater than expected by chance (0.5). As can be seen in Table 2, 5-year-old children’s voices were selected for the Positive-Affect and the Helpless items significantly greater than expected by chance. In contrast, 10-year-old children’s voices were selected for the Intelligence items significantly greater than expected by chance. Participants’ selections did not differ from chance for the Negative-Affect items.^2^

To further assess the pattern of performance, we computed two one-way analyses of variance with repeated measures on trait dimension (Positive Affect vs. Negative Affect vs. Intelligence vs. Helpless) for the proportion of participants who selected the 5-year-old voices and the reaction times. Preliminary analyses revealed no significant sex differences, as well as no significant differences between the four sets of voices used in the experiment for either of these variables. Thus, our analyses collapsed data across sex and the four sets of voices.

The analysis of variance for the proportion of participants who selected the 5-year-old voices produced a significant effect of trait dimension, *F*(2.52, 183.75) = 101.07, *p* < 0.001, η_p_^2^ = 0.58 (Helpless, *M* = 0.85 > Positive Affect, *M* = 0.62 > Negative Affect, *M* = 0.51 > Intelligence, *M* = 0.19, *p* values < 0.001, according to post-hoc Bonferroni *t*-tests). Analysis of variance of reaction times similarly yielded a significant main effect of trait dimension, *F*(3, 219) = 13.30, *p* < 0.001, η_p_^2^ = 0.15. Post-hoc Bonferroni *t*-tests (*p* < 0.001) indicated that participants took more time to process Negative-Affect items (*M* = 2478.35 ms) than items of any of the other trait dimensions, which did not differ from one another (Positive Affect, *M* = 2162.49 = Intelligence, *M* = 2069.71 ms = Helpless, *M* = 2002 ms).^3^

## Discussion

The main purpose of the present study was to shed light on the potential role that children’s voices during early childhood may play as cues to adults for the need for care, in a way that might guide caregivers’ attention and action. To that end, we presented college students with a series of voice samples of 5-year-old versus 10-year-old children and asked them to rate them on a series of trait dimensions (positive affect, negative affect, helpless, intelligence). We also measured their reactions times in doing so. We predicted, consistent with previous research examining the effect of children’s immature thinking on adult perceptions (e.g., Bjorklund et al., 2010; Hernández Blasi et al., 2015, 2017), that children with immature voices would be selected more frequently for positive affect and rated as being more helpless than children with mature voices, and that maturity of children’s voices would have no effect on negative-affect ratings. We were less certain about which children would be deemed as more intelligent. We also hypothesized that reaction times would be longer for the Negative-Affect items than for the other trait dimensions.

As for the first hypothesis, as predicted, children with immature voices (i.e., the 5-year-olds) were perceived as having greater positive affect and being more helpless than children with mature voices (i.e., the 10-year-olds). In addition, children with mature voices were deemed as higher in intelligence, whereas neither children with the immature nor mature voices were selected more often than expected by chance for the Negative-Affect items. With respect to the second hypothesis, decision-making on the Negative-Affect items was significantly the most time consuming, as predicted, reflecting the difficulty participants have making attributions of negative traits with respect to children (cf. Hernández Blasi et al., 2017).

**Fig. 2:**
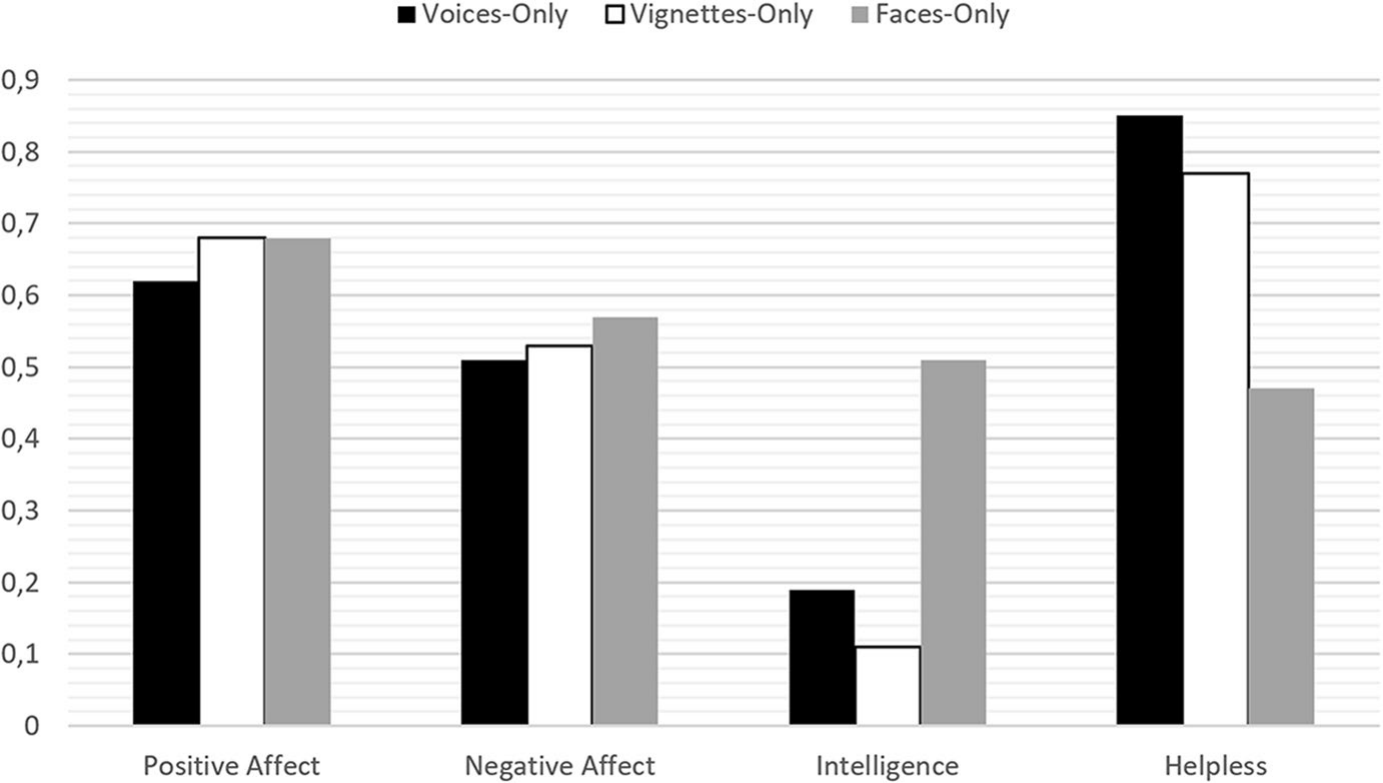
Proportion of people selecting the child with the immature voice (Voices-Only), the immature supernatural thinking (Vignettes-Only), and the immature face (Faces-Only) by trait dimension (Positive Affect, Negative Affect, Intelligence, Helpless). Voices-Only data is from the current study; Vignettes-Only and Faces-Only data from Hernández Blasi et al. (2015) for comparative purposes. Note: *t* tests on scores between .40 and .60 were not statistically different from chance in their corresponding studies; scores above .60 reflect that immature children were selected significantly greater than expected by chance; and scores below .40 indicate that mature children were selected significantly greater than expected by chance

Two important conclusions derive from the results of this study. First, acoustic features of children’s voices can provide meaningful information to adults, regardless of the content of speech, potentially important in terms of care and upbringing during early childhood. And second, when compared with results from studies assessing both cognitive cues and facial cues of preschool children (cf. Hernández Blasi et al., 2015), voices seem to be as informative for adults as cognitive cues, and more informative than faces. This is illustrated by comparing the results of the current study with those from a study examining adults’ judgments based on children’s facial features and cognitive cues (i.e., expressions of immature supernatural cognition). Figure 2 presents (1) the proportion of adults selecting the 5-year-old children’s voices in the current study and, based on data from Hernández Blasi et al. (2015), (2) the proportion of adults selecting children professing immature supernatural thinking, and (3) the proportion of adults selecting the immature face (i.e., about 5 years old versus about 10 years old) for each of the four trait dimensions. As can be seen, children with immature voices were selected for the Positive-Affect and Helpless items at similar levels, and more often than expected by chance, to children in the Hernández Blasi et al. (2015) study who expressed immature cognition. Likewise, adults selected the mature and immature children at chance levels for both voices and expressions of cognition for the Negative-Affect items, but not for Intelligence items, where the mature children were selected more often than expected by chance in both cases. In contrast, children with immature faces provoked comparable effects on adults for both cognitive and vocal cues in terms of positive affect and negative affect, but differed from the vocal and cognitive cues for the Helpless and Intelligence items, perfoming at chance levels.

Taken together, currently available research on the effects of immature cognitive, facial, and vocal cues in adults depicts a scenario where it is possible to make a preliminary proposal on the potential role of each of these cues during early development in terms of attracting potential caregivers’ attention and conveying to adults critical information about children’s needs. According to this research, faces seem to be powerful cues for caregivers during infancy (e.g., Glocker et al., 2009; Lorenz, 1943), but apparently they are not especially influential during early childhood (e.g., Luo et al., 2011, 2020), though they remain effective in arousing positive affect (e.g., Hernández & Bjorklund, 2018; Hernández Blasi et al., 2015). Conversely, cognitive cues seem to be particularly useful for caregivers during early childhood (e.g., Bjorklund et al., 2010; Periss et al., 2012), but they are not that valuable during infancy given infants’ productive language constraints. To our knowledge, the potential role of cognitive nonverbal cues in infancy to attract adults’ attention and signalling some critical information to them has not been studied in these terms. Finally, vocal cues seem to be equally worthwhile for caregivers during both infancy (e.g., through crying, as we reviewed earlier) and early childhood, as we have seen in this study. In light of this, it can be argued that vocal cues are likely one of the most reliable cues for caregivers across early development.

A number of limitations of the current study require caution in the interpretation of findings. First, whereas there is an extensive literature examining the effects on adults of infants’ and children’s facial cues (see Franklin & Volk, 2018), far fewer studies have examined the effects of infants’ and children’s nonfacial cues, particularly voices, on adults’ perceptions and behavior, and thus the results of the present study require replication and extension (although the near-identical results using simulated as opposed to children’s natural voices, reported in Appendix 1, serves as an initial replication of the present findings). Moreover, several methodological limitations of the present study must be acknowledged. (1) Our sample is mostly composed of young adults (barely 21 years old) from a WEIRD population likely without any parental experience. (2) Women were disproportionately represented in our sample, making comparisons between men and women statistically problematic. However, men and women in our study (and in the Simulated-Voices study, reported in Appendix 1) responded similarly in all conditions. Moreover, as women are (and historically have been) the primary caregivers in all societies, our results likely have ecological validity. (3) Our experimental scenario involved unknown (vs. one’s own) focal children and measured hypothetical (vs. real-life) reactions in adults, and (4) there is no distinction between the different parameters of the children’s voices, nor the degree of adults’ attention or caregiving behavior toward each voice. Future research is needed to address each of these limitations.

Indeed more research is needed on different fronts. For example, we need to know more about when in development these effects of children’s voices on adults’ reactions start. In previous research it was found, for example, that the effect of young children’s cognitive cues on adults is present in late adolescence (14–17 years old) but not before (Periss et al., 2012). Likewise, no developmental changes have been found with respect to children’s facial cues, with an identical pattern to that observed in adults being present in early adolescence (10–13 years old) (Hernández Blasi & Bjorklund, 2018; Luo et al., 2020). Nonetheless, the developmental pattern of the effects of children’s voices on adults’ perceptions is still lacking.

More research is also needed on the potential effects of different parameters of children’s voices and about the information the different parameters might convey to adults. In this study, we controlled for the maturity of children’s natural voices, but information in voices is multidimensional, and numerous different levels of information (lexical, grammatical, prosody, fluency, fundamental frequency, timbre) can potentially influence listeners’ perception of maturity of children’s voices and deserve further attention. Results in the Simulated-Voices condition described in Appendix 1 suggest, for instance, that pitch variations, as correlated with fundamental frequency, is a highly informative parameter of children’s voices. Similarly, adults in the current study might have used voice maturity as a cue to children’s age and, on that basis, made inferences about children’s intelligence or degree of helplessness. Although this is possible, children’s voices, much as their faces, may signal not only children’s age but also other features that are potentially important for parental decisions and investment (Franklin & Volk, 2018). For example, cuteness ratings based on children’s faces are highly correlated with health, age (negatively), and happiness ratings (Volk et al., 2007), and it seems likely that features in children’s voices may serve similar functions.

Another issue that needs to be addressed is the relative importance of young children’s vocal cues when either cognitive or facial cues are also available. For example, in a condition where a child with an immature voice *and* a mature face is pitted against a child with a mature voice *and* an immature face, which child, if either, would be deemed the most helpless—the one with the immature voice or the one with the immature face? In similar research contrasting young children’s cognitive and facial cues, it was found that, when these two cues were presented in a competitive manner as in the aforementioned example, cognitive cues were more influential on decisions than facial cues for both adults and older adolescents (Hernández Blasi & Bjorklund, 2018; Hernández Blasi et al., 2015). Conversely, it has been typically reported in the literature that when both facial and vocal cues are available, facial cues usually take the lead when it comes to the recognition of human emotions (e.g., Kappas et al., 1991).

Finally, even more important than disentangling which of young children’s cues (vocal, physical, and cognitive) might be more powerful and/or “honest” (in a Zahavian sense; e.g., Zahavi, 1987) is unravelling to what extent these different cues provide to caregivers redundant or complementary information about children. Multimodal signalling—that is, conveying information about an underlying trait (e.g., genetic quality) by more than one modality (e.g., faces and voices)—is common in nonhuman species (Smith et al., 2016). According to the *redundant signal hypothesis*, “multiple cues considered in combination provide a better estimate than any single cue” (Tognetti et al., 2020:824). In this sense, for example, available research on young children’s signalling suggests that vocal, facial, and cognitive cues are likely redundant regarding positive affect. However, when it comes to signalling young children’s helplessness, only vocal and cognitive cues, but not faces, seem to be redundant. Interestingly enough, on the basis of cognitive psychology, clinical neuroscience, and neuroimaging evidence, it seems that in humans some of these systems (e.g., faces and voice processing) develop in parallel, with audiovisual integration of information taking place simultaneously (e.g., Belin et al., 2004, 2011). This parallel development may indicate that these different signalling systems might have evolved in parallel as well. The integration of the information provided by these systems may become critical because “it allows our brain to exploit redundancies between face and voice and combine non-redundant, complementary cues to maximize information gathered from the two modalities” (Belin et al., 2011:719). We do not yet know to what extent children’s vocal, facial, and cognitive cues operate during early childhood as “back up” or redundant signals (conveying similar information about young children), or as “multiple messages” or complementary signals (each cue conveying different information about young children). This is undoubtedly a main challenge for researchers in the near future, and an undertaking that is worth the effort.

To our knowledge this is the first study to report evidence about how children’s voices can influence adults’ reactions toward young children in terms of positive affect, negative affect, intelligence, and helpless appraisals. This study has shown that children with immature voices are perceived more positively and deemed as more helpless and less intelligent than children with mature voices. Overall, this study draws attention to the fact that voice, regardless of the content of speech, is a powerful cue for children’s caregivers, not just during infancy but also during early childhood, becoming one of the richest sources of information for parents and others during the first six years of life.

## Figures and Tables

**Fig. 3:**
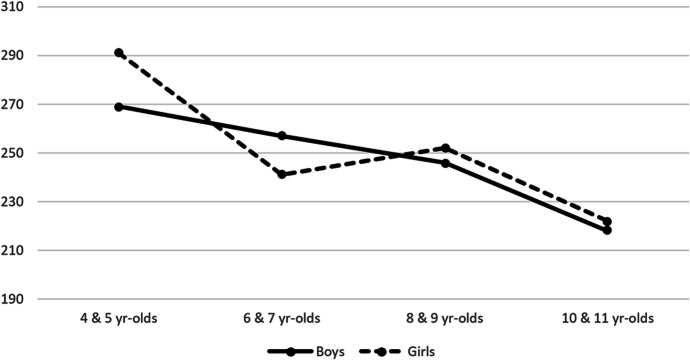
Mean pitch (fundamental frequency, in Hz) for a sample of 42 4- to 11-year-old children (22 boys, 20 girls) when verbalizing a neutral-content sentence (“I like the beach more than the mountains”)

## Appendix 1

### Simulated-Voices Condition

In this condition, we presented samples of immature and mature simulated voices of real 7- to 8-year-old children, manipulated to resemble the voices of preschool and school-age children, respectively. These simulations were made by artificially adjusting, through a voice editor, the original fundamental frequency of the real 7- to 8-year-old children’s voices—also verbalizing neural content sentences—to the typical fundamental frequency of either 5-year-old or 10-year-old children. With the Simulated-Voices condition we aimed to create an experimentally more reliable condition wherein, and different from the Natural-Voices condition, most voice parameters other than pitch, as expressed by mean fundamental frequency (e.g., timbre, intonation, speech rate, pauses; see Titze, 2000, for a more detailed description), were the same for each pair of preschool and school-age children.

For this condition, the recordings of four boys and four girls (all 7 or 8 years old) were selected, and four different sets for testing were generated. For each child, we created both a more immature and a more mature version of the four neutral sentences using the audio-editor Audacity. The immature version of each child’s voice was intended to resemble as much as possible the voice of a 4- to 7-year-old child, and the mature version, the voice of an 8- to 10-year-old child. After experimenting with different voice parameter changes and samples of children’s voices of different ages, we found that modifications of about ± 10% of the pitch (fundamental frequency, measured in Hz) of 7- to 8-year-old children was the most parsimonious and convincing way of obtaining these effects. Once we had simulated and adjusted the immature and the mature voices for every child, we generated the respective four sets of sentences. As in the Natural-Voices condition, every set contained four pairs of voices (half from boys, half from girls) verbalizing the four neutral sentences (each pair verbalized a single sentence per set). However, this time only two boys’ and two girls’ immature and mature pairs of voices were included in each set, so that overall each child appeared in only two sets (not in four), sometimes articulating the same sentence, sometimes a different one, than in the previous set. As in the Natural-Voices condition, all voices were equalized to about 72 dB volume. (Table

**Table 3:** Mean pitch (fundamental frequency, in Hz) of the boys’ and girls’ simulated voices across the four neutral sentences verbalized in every set. (standard deviations in parenthesis)

	Boys	Girls
Immature	273.07 (13.33)	282.37 (24.48)
Mature	227.00 (11.83)	240.75 (19.39)

3 presents mean pitch details for immature and mature boys’ and girls’ voices across the four sets.)

The sample for this study was composed of 72 adults (58 female, *M*_age_ = 22.4 years, *SD* = 6.5 years, age range = 17–48 years), all college students, most taking classes in psychology (58, 81%), attending the same public urban university in eastern Spain as participants in the Natural-Voices condition, and with the same socioeconomic background. Main results can be seen in Table

**Table 4:** Proportion of participants selecting the child with the immature voice, and mean reaction time (in milliseconds) by trait dimension (Positive Affect, Negative Affect, Intelligence, Helpless) (standard deviations in parenthesis)

	Positive Affect (*n* = 4)	Negative Affect (*n* = 4)	Intelligence (*n* = 2)	Helpless (*n* = 3)
Proportion	.81^*a*^ (.16)	.44 (.23)	.35^*b*^ (.24)	.71^*a*^ (.23)
Reaction Time	2017.16 (587.08)	2464.58 (556.58)	2299.43 (764.65)	2284.06 (660.61)

^*a*^ selecting the child with the immature voice significantly greater than expected by chance; ^*b*^ selecting the child with the mature voice significantly greater than expected by chance. Significance set at *p* < .001

^4^. As in the data for the Natural-Voices condition, the simulated voices of 5-year-old children were selected significantly more often than expected by chance for the Positive-Affect and Helpless traits, whereas the simulated voices of the 10-year-old children were selected for the Intelligence trait significantly more often than expected by chance. Again, as in the analyses of the natural voices, selections did not differ from chance for the Negative-Affect items.

A one-way analysis of variance with repeated measures on the proportion of participants who selected the child with the immature voice produced a significant effect of trait dimension, *F*(2.54, 180.55) = 62.05, *p* < 0.001, *η*_p_^2^ = 0.47 (Positive Affect, *M* = 0.81 > Helpless, *M* = 0.71 > Negative Affect, *M* = 0.44 > Intelligence, *M* = 0.35, *p* values ≤ 0.007, according to post-hoc Bonferroni *t*-tests, *p* < 0.001). A one-way ANOVA of reaction times also yielded a significant effect of trait dimension, *F*(2.47, 175.46) = 11.40, *p* < 0.001, η_p_^2^ = 0.14. Post-hoc Bonferroni *t*-tests (*p* values ≤ 0.03) showed that participants took more time to process Negative-Affect items (*M* = 2464.58 ms) than Helpless and Positive-Affect items (Helpless, *M* = 2284.06 > Positive Affect, *M* = 2017.16 ms). Intelligence items (*M* = 2299.43 ms) took more time to be processed than Positive-Affect items, but they did not differ significantly from items of the other two trait dimensions.

## Appendix 2

### Criteria for Selecting Vocal Stimuli

#### Internal Criteria

In order to make a primary selection from our available sample of 5- and 10-year-old children’s recorded voices (for the Natural-Voices condition) and 7- to 8-year-old children’s recorded voices (for the Simulated-Voices condition), two teams of researchers, independently of each other, made assessments as to which children’s voices to discard, and why, on the basis of the first neutral sentence recorded (“I like the beach more than the mountain”). The research teams made the final decision to discard one 5-year-old, five 7- to 8-year-olds, and one 10-year-old, based on three criteria: (1) substantial deviation from the typical pitch range for the corresponding age group; (2) children’s pronunciation problems or lack of voice sharpness; and/or (3) too high background noise.

#### External Criteria

We asked four full-time colleagues from the Department of Psychology (two males and two females) to listen individually and sequentially to the series of 24 children’s voice samples selected for our study. Sixteen of the voices belonged to the Simulated-Voices condition: 8 immature and 8 mature versions of our eight 7- to 8-year-old selected children. The other 8 belonged to the Natural-Voices condition, four 5- and four 10-year-olds. All children verbalized the first neutral sentence mentioned above. The 24 children’s voice samples were presented in random prearranged order. Our colleagues were specifically asked to rate each sentence regarding: (a) background noise level (*high, medium, low*); (b) sharpness/clearness of voice (*good, so-so, bad*); (c) the probable age of the child (> *7 years old;* ≤ *7 years old; I don´t know*); and (d) the probable sex of the child (*boy; girl; I don´t know*).

On average, 91% of the eight children’s voices from the Natural-Voices condition were found to be “good” on sharpness; 88% were found to have a “low” background noise level; 88% were positively identified regarding age; and 72% were positively identified regarding sex. (Especially for young children, boys’ and girls’ natural voices are typically difficult to distinguish.) For the Simulated-Voices condition, 99% of the 16 children’s voices were found to be “good” on sharpness; 89% were found to have a “low” background noise level; 80% were positively identified regarding age; and 48% were positively identified regarding sex.

Overall we deemed as satisfactory the results of this external criteria assessment. However, we judged the percentage of positive sex identifications of the children’s voices in the Simulated-Voices condition to be below expectation (below 50%). In this vein, we found that the four experts agreed on the difficulty of distinguishing the sex of the same three girls’ mature voices (often mistaken as boys), and three of the four agreed on difficulties determining the sex of the same two girls’ immature voices. Therefore we decided to re-edit these five children’s voices across the four neutral sentences used in the study, looking for a higher pitch, a typical trait of girls’ voices, particularly on the mature voices; the only self-imposed limitation to those changes was that the new voices did not sound unnatural (i.e., metallic or distorted).

As a result, overall about 70% of voices in the Simulated-Voices condition could be generated through a 10 ± 2% Hz change on the original 7- to 8-year-old children’s voice pitch, as expressed by fundamental frequency, whereas about 30% of the voices entailed changes less than 6% or greater than 12% Hz. Mean percentage of the pitch changes performed on the 16 pairs of voices used across the four sets of the Simulated-Voices condition was 9.8% (*SD* = 5.2) Fig. 3 presents a profile of children’s pitch development from 4 to 11 years of age, based on the original pitch measures on the first neutral sentence recorded (“I like the beach more than the mountains”). This information provides an idea about how much the profile of the voices in the Simulated-Voices condition resembled the profile of the real voices’ pitch.

## Footnotes

1 We also ran a Simulated-Voices condition (*N* = 72, 58 females) based on pitch (fundamental frequency) manipulations; because the results were nearly identical to those obtained using natural voices, we report it in Appendix 1.

2 Highly similar results were found when simulated voices were used as stimuli (Appendix 1), both for proportion of participants selecting the child with the immature voice (Positive Affect = .81; Negative Affect = .44; Intelligence = .35; and Helpless = .71) and for reaction times (Positive Affect = 2017.16 ms; Negative Affect = 2464.58 ms; Intelligence = 2299.43 ms; and Helpless = 2284.06 ms).

3 Three trait dimensions in each ANOVA did not satisfy the normality assumption. However, ANOVA tests are very robust to violations (Langdridge & Hagger-Johnson, 2009:293). Indeed results were identical when applying Friedman non-parametric tests. We applied Greenhouse–Geisser corrections when sphericity assumption was violated (here, for the proportion of participants who selected the 5-year-old voices).

## References

[CR1] Assmann, P., Barreda, S., & Nearey, T. (2011). Perception of speaker sex in children’s voices. *Proceedings of Meetings on Acoustics 162ASA,* 14, 060009. Acoustical Society of America. 10.1121/1.4793571

[CR2] Assmann, P., Barreda, S., & Nearey, T. (2013). Perception of speaker age in children’s voices. *Proceedings of Meetings on Acoustics ICA2013,* 19, 060059. Acoustical Society of America. 10.1121/1.4800918

[CR3] Assmann PF, Kapolowicz MR, Barreda S (2018). Perception of talker height and sex from children’s voices. The Journal of the Acoustical Society of America.

[CR4] Balasubramaniam RK, Nikhita N (2017). Voice mutation during adolescence in Mangalore, India: Implications for the assessment and management of mutational voice disorders. Journal of Voice.

[CR5] Belin, P. (2011). “Hearing voices”: Neurocognition of the human voice. In J. Decety & J. T. Cacciopo (Eds.), *The Oxford handbook of social neuroscience*. 10.1093/oxfordhb/9780195342161.013.0025

[CR6] Belin P, Bestelmeyer PE, Latinus M, Watson R (2011). Understanding voice perception. British Journal of Psychology.

[CR7] Belin P, Fecteau S, Bédard C (2004). Thinking the voice: Neural correlates of voice perception. Trends in Cognitive Sciences.

[CR8] Berger, T., Peschel, T., Vogel, M., Pietzner, D., Poulain, T., Jurkutat, A., ... & Fuchs, M. (2019). Speaking voice in children and adolescents: Normative data and associations with BMI, Tanner stage, and singing activity. *Journal of Voice*, *33*, 580.e21-580.e30. 10.1016/j.jvoice.2018.01.006 10.1016/j.jvoice.2018.01.006 29807693

[CR9] Bjorklund DF, Hernández Blasi C, Periss VA (2010). Lorenz revisited: The adaptive nature of cognitive immaturity. Human Nature.

[CR10] Borgi M, Cogliati-Dezza I, Brelsford V, Meints K, Cirulli F (2014). Baby schema in human and animal faces induces cuteness perception and gaze allocation in children. Frontiers in Psychology.

[CR11] Bowlby, J. (1969). *Attachment and loss: Vol. 1. Attachment*. New York: Basic Books.

[CR12] Bustos I (2012). *La voz: **L**a técnica y la expresión* [Voice: Technique and expression].

[CR13] Cappellari VM, Cielo CA (2008). Vocal acoustic characteristics in pre-school aged children. Brazilian Journal of Otorhinolaryngology.

[CR14] Chittora A, Patil HA (2017). Data collection of infant cries for research and analysis. Journal of Voice.

[CR15] Cook ND (2002). Tone of voice and mind: The connections between intonation, emotion, cognition and consciousness.

[CR16] De Vries MW (1984). Temperament and infant mortality among the Masai of East Africa. American Journal of Psychiatry.

[CR17] de Waal FBM (1996). Good natured: The origins of right and wrong in humans and other animals.

[CR18] Dettwyler KA, Stuart-Macadam P (2017). A time to wean: The hominid blueprint for the natural age of weaning in modern human populations. Breastfeeding: Biocultural perspectives.

[CR19] Franklin P, Volk A (2018). A review of infants’ and children’s facial cues’ influence on adults’ perceptions and behaviors. Evolutionary Behavioral Sciences.

[CR20] Frodi A, Lester B, Boukydis C (1985). When empathy fails: Aversive infant crying and child abuse. Infant crying.

[CR21] Fullard W, Reiling AM (1976). An investigation of Lorenz’s “babyness”. Child Development.

[CR22] Furlow FB (1997). Human neonatal cry quality as an honest signal of fitness. Evolution and Human Behavior.

[CR23] Glocker ML, Langleben DD, Ruparel K, Loughead JW, Gur RC, Sachser N (2009). Baby schema in infant faces induces cuteness perception and motivation for caretaking in adults. Ethology.

[CR24] Goetz JL, Keltner D, Simon-Thomas E (2010). Compassion: An evolutionary analysis and empirical review. Psychological Bulletin.

[CR25] Grossman T, Oberecker R, Koch SP, Friederici AD (2010). The developmental origins of voice processing in the human brain. Neuron.

[CR26] Gustafsson E, Levréro F, Reby D, Mathevon N (2013). Fathers are just as good as mothers at recognizing the cries of their baby. Nature Communications.

[CR27] Hauser MD (1996). The evolution of communication.

[CR28] Hernández Blasi C, Bjorklund DF (2018). Adolescents’ sensitivity to children’s supernatural thinking: A preparation for parenthood?. Psicothema.

[CR29] Hernández Blasi C, Bjorklund DF, Ruiz Soler M (2015). Cognitive cues are more compelling than facial cues in determining adults’ reactions towards young children. Evolutionary Psychology.

[CR30] Hernández Blasi C, Bjorklund DF, Ruiz Soler M (2017). Children’s supernatural thinking as a signalling behaviour in early childhood. British Journal of Psychology.

[CR31] Kappas A, Hess U, Scherer KR, Feldman RS, Rimé B (1991). Voice and emotion. Fundamentals of nonverbal behavior.

[CR32] Karlsson I, Rothenberg M (1987). Sex differentiation cues in the voices of young children of different language background. The Journal of the Acoustical Society of America.

[CR33] Konner M (2010). The evolution of childhood: Relationships, emotion, mind.

[CR34] Kringelbach ML, Stark EA, Alexander C, Bornstein MH, Stein A (2016). On cuteness: Unlocking the parental brain and beyond. Trends in Cognitive Sciences.

[CR35] Lancaster JB, Altmann J, Sherrod LR, Rossi A (2010). Parenting across the life span: Biosocial dimensions.

[CR36] Lancy DF (2015). The anthropology of childhood: Cherubs, chattel, changelings.

[CR37] Lang SF, Fowers BJ (2019). An expanded theory of Alzheimer’s caregiving. American Psychologist.

[CR38] Langdridge D, Hagger-Johnson G (2009). Introduction to research methods and data analysis in psychology.

[CR39] Lorenz KZ (1943). Die angeboren Formen moglicher Erfahrung [The innate forms of possible experience]. Zeitschrift für Tierpsychologie.

[CR40] Luo LZ, Li H, Lee K (2011). Are children’s faces really more appealing than those of adults? Testing the baby schema hypothesis beyond infancy. Journal of Experimental Child Psychology.

[CR41] Luo L, Ma X, Zheng X, Zhao W, Xu L, Becker B, Kendrick KMF (2015). Neural systems and hormones mediating attraction to infant and child faces. Frontiers in Psychology.

[CR42] Luo L, Zhang Q, Wang J, Lin Q, Zhao B, Xu M, Langley C, Li H, Gao S (2020). The baby schema effect in adolescence and its difference from that in adulthood. Journal of Experimental Child Psychology.

[CR43] Mampe B, Friederici AD, Christophe A, Wermke K (2009). Newborns’ cry melody is shaped by their native language. Current Biology.

[CR44] Michelsson K, Michelsson O (1999). Phonation in the newborn, infant cry. International Journal of Pediatric Otorhinolaryngology.

[CR45] Periss V, Hernández Blasi C, Bjorklund DF (2012). Cognitive “babyness”: Developmental differences in the power of young children’s supernatural thinking to influence positive and negative affect. Developmental Psychology.

[CR46] Perry TL, Ohde RN, Ashmead DH (2001). The acoustic bases for gender identification from children’s voices. The Journal of the Acoustical Society of America.

[CR47] Rosenfield KA, Sorokowska A, Sorokowski P, Puts DA (2020). Sexual selection for low male voice pitch among Amazonian forager-horticulturists. Evolution and Human Behavior.

[CR48] Schneider B, Zumtobel M, Prettenhofer W, Aichstill B, Jocher W (2010). Normative voice range profiles in vocally trained and untrained children aged between 7 and 10 years. Journal of Voice.

[CR49] Senese VP, De Falco S, Bornstein MH, Caria A, Buffolino S, Venutti P (2013). Human infant faces provoke implicit positive affective responses in parents and non-parents alike. PLoS ONE.

[CR50] Sergeant DC, Sjölander PJ, Welch GF (2005). Listeners’ identification of gender differences in children’s singing. Research in Music Education.

[CR51] Seyfarth RM, Cheney DL (2003). Signalers and receivers in animal communication. Annual Review of Psychology.

[CR52] Smith HM, Dunn AK, Baguley T, Stacey PC (2016). Concordant cues in faces and voices: Testing the backup signal hypothesis. Evolutionary Psychology.

[CR53] Soltis J (2004). The signal functions of early infant crying. Behavioral and Brain Sciences.

[CR54] Titze, I. R. (2000). *Principles of voice production* (second printing). Iowa City, IA: National Center for Voice and Speech.

[CR55] Titze IR (2017). Human speech: A restricted use of the mammalian larynx. Journal of Voice.

[CR56] Tognetti A, Durand V, Barkat-Defradas M, Hopfensitz A (2020). Does he sound cooperative? Acoustic correlates of cooperativeness. British Journal of Psychology.

[CR57] Trivers RL (1974). Parent-offspring conflict. American Zoologist.

[CR58] Trollinger VL (2003). Relationships between pitch-matching accuracy, speech fundamental frequency, speech range, age, and gender in American English-speaking preschool children. Journal of Research in Music Education.

[CR59] Volk AA, Lukjanczuk JL, Quinsey VL (2007). Perceptions of child facial cues as a function of child age. Evolutionary Psychology.

[CR60] Warneken F, Tomasello M (2006). Altruistic helping in human infants and young chimpanzees. Science.

[CR61] Wermke, K., Ruan, Y., Feng, Y., Dobnig, D., Stephan, S., Wermke, P., ... & Shu, H. (2017). Fundamental frequency variation in crying of Mandarin and German neonates. *Journal of Voice*, *31*, 255.e25-255.e30. 10.1016/j.jvoice.2016.06.009 10.1016/j.jvoice.2016.06.009 27397111

[CR62] Wermke, K., Teiser, J., Yovsi, E., Kohlenberg, P. J., Wermke, P., Robb, M., ... & Lamm, B. (2016). Fundamental frequency variation within neonatal crying: Does ambient language matter? *Speech, Language and Hearing*, *19*, 211-217. 10.1080/2050571X.2016.1187903

[CR63] Wolff, P. H. (1969). The natural history of crying and other vocalizations in early infancy. In B. M. Foss (Ed.), *Determinants of infant behavior* (Vol. 4) (pp. 81–111). London: Methuen.

[CR64] Zahavi, A. (1987). The theory of signal selection and some of its implications. In V. P. Delfino (Ed.), *International symposium of iological evolution* (pp. 305–327). Bari, Italy: Adriatica Editrice.

